# Design of Transmission-Type Refractive Index Sensor, Based on Silica Planar Lightwave Circuit Using Combination of Refractive Angle and Phase Measurements

**DOI:** 10.3390/s19194095

**Published:** 2019-09-22

**Authors:** Koichi Maru

**Affiliations:** Faculty of Engineering and Design, Kagawa University, 2217-20 Hayashi-cho, Takamatsu, Kagawa 761-0396, Japan; maru@eng.kagawa-u.ac.jp; Tel.: +81-87-864-2230

**Keywords:** sensing, waveguide devices, refractive index measurements, planar lightwave circuit technology, interferometry

## Abstract

A transmission-type refractive index sensor, based on planar lightwave circuit (PLC) technology is proposed. In the proposed structure, we introduce a combination of coarse measurements, using the dependence of the angle of refraction and fine measurement, and the dependence of the phase on the refractive index to measure the absolute refractive index precisely, without expensive optical measurement equipment. The theoretical model of the proposed refractive index sensor is derived based on Fourier optics and transfer function to simulate its performance. The simulation results for the use of the 2.5%-Δ silica-based PLC technology indicate that the proposed structure has the potential to achieve a refractive index error of approximately 1 × 10^−6^ RIU or less when a monitored power deviation of ±0.05 dB is accepted.

## 1. Introduction

Refractive index measurements are required in numerous applications, including chemical analysis, biochemistry, and industrial fields. To meet the demands, optical sensing methods have been developed for the direct and precise measurement of the refractive index. Several techniques for refractive index measurement, including the minimum deviation-based method [1,2], critical angle method [3,4], use of Fabry-Perot interferometers [5], surface plasmon resonance (SPR) [6,7,8], resonance in photonic crystals [9], fiber Bragg gratings [10,11,12], and multimode fibers [13] have been proposed. In most of these methods, expensive optical measurement equipment, such as an optical spectrum analyzer (OSA) is required in addition to a sensing unit, in order to measure the spectrum of the light from the sensing unit. As the accuracy highly depends on the equipment, these methods continue to face the challenge of measuring the refractive index with high accuracy (10^−6^ RIU or less). The limitation of the precision of the equipment is also a challenge. Furthermore, a compact sensor is desired for practical use. However, most of the above methods are based on bulk optics. To overcome these problems, we previously proposed a reflection-type integrated refractive index sensor [14]. This sensor was based on planar lightwave circuit (PLC) technology [15,16], which is widely used for optical passive devices deployed in optical communication systems. In the proposed structure, the combination of coarse measurement utilizing the dependence of the angle of refraction, and fine measurement utilizing the dependence of the phase on the refractive index, were introduced to precisely measure the absolute refractive index. The proposed sensor did not require expensive optical measurement equipment, such as an OSA. However, the previous work was limited to only a preliminary phase and quantitative evaluation was not performed. Furthermore, in conventionally proposed reflection-type devices, the performance can be degraded if the reflecting edge is marginally fluctuated.

In this paper, we propose a transmission-type refractive index sensor based on the PLC technology. The use of a waveguide device enables us to realize a compact sensor that is preferable for practical use. We describe the principle of the proposed method and the design of the integrated optical circuit. The performance of the proposed sensor is simulated using a theoretical model.

## 2. Principle

**Figure 1:**
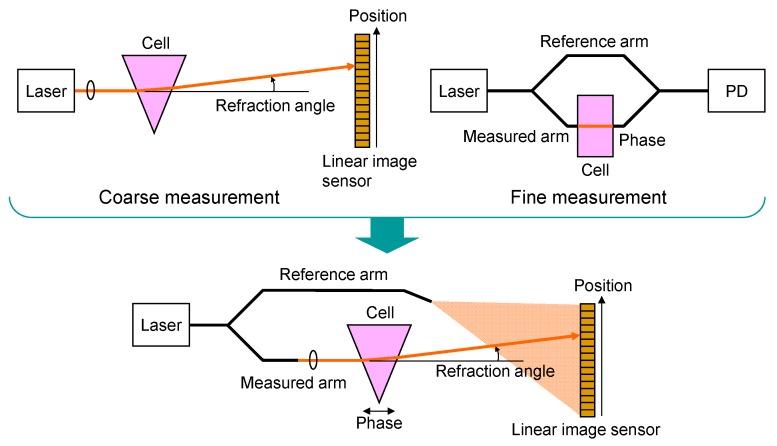
Concept of proposed refractive index sensor.

Figure 1 illustrates the concept of the proposed refractive index sensor. In this method, coarse measurements, based on the dependence of the refraction angle, and fine measurement based on the dependence of the phase on the refractive index of the material (liquid) under test, are combined. The absolute value of the refractive index is measured by measuring the refraction angle after the propagation of the wedge-shaped cell, filled with the liquid to be tested. However, the size must be larger for higher resolutions to measure the refraction angle more precisely. High resolution can also be readily obtained by measuring the phase change of the beam passing through the cell, based on interferometry. In this technique, however, the absolute value of the refractive index is difficult to measure over a wide index range because the phase folds into a period of 2π rad. The proposed method combines the advantages of these two techniques to measure the absolute refractive index precisely using a Mach-Zehnder interferometer. One beam traverses the wedge-shaped cell and is focused before the linear image sensor where CMOS image sensors, charge-coupled devices (CCDs), or photodiodes (PDs) are lined. Another beam propagates a reference arm and spreads before the linear image sensor. The absolute value of the refractive index of the material under test is roughly measured as the position of the focusing beam, which is dependent of the refractive angle at the cell. Simultaneously, the phase of the beam, passing through the wedge-shaped cell, is measured as the power of the output signal using the interference between the focusing beam and spread beam from the reference arm. Then, the relative refractive index is finely estimated from the phase. By combining the information regarding the beam position and phase, the absolute value of the refractive index can be precisely measured. This method does not require expensive optical measurement equipment and is capable of realizing real-time measurements.

**Figure 2:**
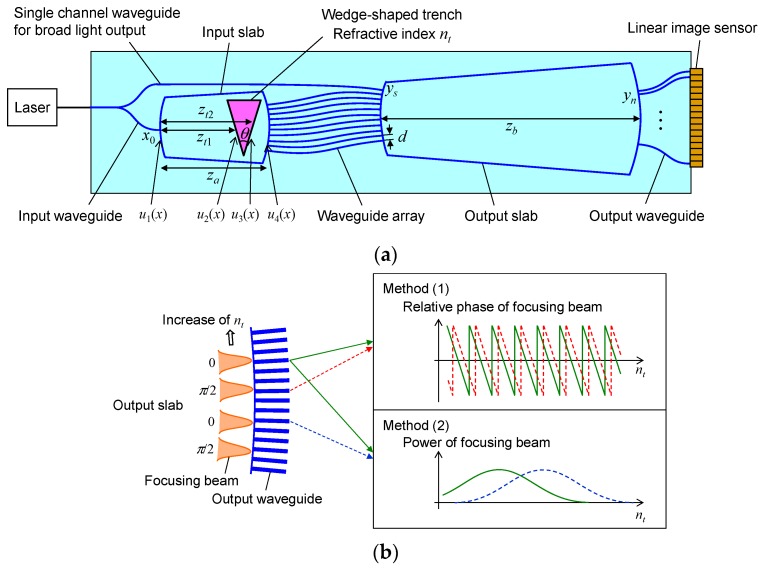
Configuration of PLC-based transmission-type refractive index sensor. (**a**) Circuit layout. (**b**) Methods for obtaining accurate information regarding phase and focusing position.

The proposed concept is based on Mach-Zehnder interferometry. The waveguide-based interferometer has advantages compared to the interferometer, which is based on bulk optics, as it can be compact and stable from environmental disturbances. This is essential for the precise measurements of the phase. In this paper, the use of PLC technology is supposed to be used. Figure 2 depicts the configuration of the PLC-based transmission-type refractive index sensor. The light from the laser is split into two beams. One beam is input to the measured arm consisting of an arrayed waveguide grating (AWG). A wedge-shaped trench is formed in the input slab of the AWG and filled with the material under test. The beam input to the input slab passes through and is refracted at the trench, and is coupled to the waveguide array. The light traversing the waveguide array is spatially filtered, phase-shifted, and output to the output slab. In the output slab, the light is interfered, focused at the end of the output slab, and coupled to the output ports consisting of output channel waveguides. The second beam is input to the reference arm consisting of a single channel waveguide directly connected to the output slab. The beam from the reference arm is spread into the output slab and interfered with the focusing beam. By monitoring the output power from the output waveguides, both the position and phase of the focusing beam is measured, and finally the absolute value of the refractive index of the material under test is estimated.

To obtain accurate information regarding the phase and focusing position, the following methods are introduced (schematically presented in Figure 2b):

- Method (1): Two focusing beams are formed at the output side of the output slab to monitor the phase of the light propagating through the trench, using the quadrature detection technique [17,18,19]. To determine the phase by the quadrature detection, the relative phases of the two focusing beams to the broad beam are adjusted to have a difference of *π*/2 rad at the output side of the output slab, and the phase of the light, propagating through the trench, is estimated from the output power interfered with the broad beam. One simple method to obtain two focusing beams with a π/2 relative phase difference is to use two input waveguides with a relative phase difference of *π*/2. In this method, however, the phase difference depends on the refractive index of the material in the trench, which results in a difficult phase estimation issue because the optical paths of the light in the trench from the two input waveguides are not strictly the same. To avoid this problem, an alternative approach, using only one input waveguide, is introduced. In this method, the waveguide array is used as a spatial filter, i.e., the amplitude and phase distribution of the waveguide array is designed such that two focusing beams whose relative phases differ by π/2 are constructed from the output beams from the waveguide array.
- Method (2): If one intends to increase the phase change with the refractive index change of the material in the trench to increase the measurement sensitivity to the refractive index, the shift of the focusing beam at the end of the output slab becomes excessively small, such that the light is observed from only the same output port, even if the phase change of the light propagating through the trench is large. Consequently, the position of the focusing beam cannot be precisely measured, which makes it difficult to measure the absolute refractive index. To overcome this problem, a configuration of differential monitoring for the beam position is introduced. In this configuration, an additional set of focusing beams consisting of two focusing beams, each of which corresponds to each of the two original focusing beams described in Method (1), is provided, and the four focusing beams are used jointly. The original focusing beam and additional focusing beam are designed to have an in-phase relation, i.e., the relative phase of the additional focusing beam to the broad beam is set to the same as that in the corresponding original focusing beam. The focusing positions are set such that the position of each additional focusing beam approaches an output waveguide, when the position of the corresponding original focusing beam is separating from another output waveguide, according to the change in the refractive index of the material in the trench. By comparing the output power corresponding to the original beams and power corresponding to the additional beams, the absolute refractive index depending on these focusing positions can be roughly estimated.

By combining these methods, i.e., the fine measurement of the relative refractive index by Method (1) and coarse measurement of the absolute refractive index by Method (2), it is possible to measure the absolute value of the refractive index precisely.

## 3. Theoretical Model

We derived the theoretical model of the proposed refractive index sensor based on Fourier optics and transfer function [20,21,22], which have been widely used for simulating characteristics of various AWG-based waveguide devices, to analyze its performance. Suppose that *z_a_* and *z_b_* are the focal lengths of the input, and output slabs, respectively, *n_s_* and *k_s_* = 2*πn_s_*/*λ* are the effective refractive index and propagation constant of the guided mode of the slabs, where *λ* is the wavelength, *d* is the pitch of the waveguide array at the edge of the slabs, *x*_0_ is the connecting position of the input waveguide at the input slab, *y_n_* is the connecting position of the *n*th output waveguide at the output slab, and *x* and *y* are the coordinates on the edges of the input and output slabs. In the input slab, the wedge-shaped trench with an opening angle of *θ* is formed and filled with the liquid under testing with a refractive index of *n_t_*. The distances of the input-side edge and output-side edge of the trench from the input side of the input slab are *z_t_*_1_ and *z_t_*_2_ along the center axis of the slab, respectively.

### 3.1. Input Slab

The model of the propagation in the input slab is derived using the Fresnel approximation [23]. The input field *u*_1_(*x*) is given by,

(1) $$u_{1}(x)=E_{0}u_{in}\left(x-x_{0}\right),$$

where *u_in_*(*x* − *x*_0_) is the input mode field of the input waveguide at the input to the slab and *E*_0_ is the complex amplitude of the light from the input waveguide. The field distribution before the trench, *u*_2_(*x*), is given by the two-dimensional Fresnel diffraction of *u*_1_(*x*) as:

(2) $$u_{2}(x)=\sqrt{\frac{jk_{s}}{2\pi z_{t1}}}e^{-jk_{s}z_{t1}}u_{1}(x)*g_{t1}(x).$$

Here, *g_t_*_1_(*x*) = exp(−*jk_s_x*^2^/(2*z_t_*_1_)). The symbol ‘*’ represents the convolution of the non-periodical functions.

The phase of the light is shifted and inclined with *k_s_x*Δtan*θ* after propagating the trench, where Δ = (*n_s_* − *n_t_*)/*n_s_*. The field of the light after propagating the trench, *u*_3_(*x*), is given by,

(3) $$u_{3}(x)=\frac{jk_{s}}{2\pi }\sqrt{\frac{1-\Delta }{z_{t1}z_{t2t1}}}e^{-jk_{s}(z_{t1}+z_{t2t1}(1-\Delta ))}e^{jk_{s}x\Delta \tan\theta }u_{1}(x)*g_{t1}(x)*g_{w}(x),$$

where *z_t_*_2*t*1_ = *z_t_*_2_ − *z_t_*_1_, *z_w_* = (*n_s_*/*n_t_*)*z_t_*_2*t*1_, and *g_w_*(*x*) = exp(−*jk_s_x*^2^/(2*z_w_*)).

The field distribution before the waveguide array, *u*_4_(*x*), is given by the two-dimensional Fresnel diffraction of *u*_3_(*x*). Supposing that the input edge of the waveguide array is arranged on the arc of the output side of the input slab and the *n_t_* value is near *n_s_*, *u*_4_(*x*) is approximately derived after mathematical manipulation as,

(4) $$u_{4}(x)≅\frac{\hat{k}(\Delta )}{2\pi }e^{jk_{s}\frac{z_{t2}x\Delta \tan\theta }{z_{a}}}\int_{-\infty }^{\;\infty }u_{1}(x^{\prime })e^{jk_{s}\frac{(x+z_{at2}\Delta \tan\theta )x^{\prime }}{z_{a}}}dx^{\prime },$$

(5) $$\hat{k}(\Delta )=\left(1-\frac{z_{t2t1}}{2z_{a}}\Delta \right)\sqrt{\frac{j2\pi k_{s}}{z_{a}}}e^{jk_{s}\left(-z_{a}+z_{t2t1}\Delta +\frac{z_{at2}z_{t2}\Delta^{2}\tan^{2}\theta }{2z_{a}}\right)},$$

where *z_at_*_2_ = *z_a_* − *z_t_*_2_.

### 3.2. Waveguide Array

The light after propagating through the input slab is coupled to the waveguide array consisting of 2*I* + 1 channel waveguides. The amplitude of the light coupled to the *i*th waveguide in the waveguide array, *P_i_*, is expressed with the overlap integral between *u*_4_(*x*) and the mode field of the *i*th waveguide at the input slab-array interface, *u_a_*(*x* − *id*), and derived as:

(6) $$P_{i}(\Delta )=\hat{k}(\Delta )\sqrt{\frac{z_{a}}{j2\pi k_{s}}}f_{a}(x_{0}+z_{t2}\Delta \tan\theta )E_{0}e^{jk_{s}\left(z_{a}+\frac{x_{0}z_{at2}\Delta \tan\theta +id(x_{0}+z_{t2}\Delta \tan\theta )}{z_{a}}\right)}\int_{-\infty }^{\;\infty }u_{in}(x^{\prime })e^{jk_{s}\frac{(z_{at2}\Delta \tan\theta +id)x^{\prime }}{z_{a}}}dx^{\prime }.$$

Here, the image field of one waveguide in the waveguide array produced on the input coordinate *x* with the Fraunhofer diffraction is denoted by *f_a_*(*x*), which is a slowly changing function with respect to *x* in comparison with *u_a_*(*x* − *id*).

The phase of the light, coupled with the *i*th waveguide, is relatively shifted by 2*πf/δf_FSR_* after the light propagates through the array, where *f* is the frequency of the light and *δf_FSR_* is the free spectral range of the waveguide array. The field distribution produced on the interface between the array and output slab, *E_b_*(*y’*, Δ), is given by,

(7) $$E_{b}(y^{\prime },\;\Delta )=\sum_{i=-I}^{I}u_{b}(y^{\prime }-id)P_{i}(\Delta )A_{i}e^{j2\pi i\frac{f}{\delta f_{FSR}}},$$

where *u_b_*(*y’* − *id*) is the mode field of the *i*th waveguide at the output slab-array interface and *y’* represents the coordinates on the interface. *A_i_* is the complex transmitting coefficient of the *i*th waveguide array, which expresses the amplitude and phase distribution of the waveguide array functioned as a spatial filter.

### 3.3. Output Slab

The field at the output side of the output slab is derived in a similar manner to our previous work, based on Fourier optics [24]. The light from the waveguide array traverses through the output slab and is focused on the interface between the output slab and output waveguides. The field distribution of the focusing light, *E^b^_out_*(*y*, Δ), is derived as the Fourier transform of *E_b_*(*y’*, Δ). Supposing that the input mode field function *u_in_*(*x*) is sufficiently narrow, *E^b^_out_*(*y*, Δ) is given by,

(8) $$E_{out}^{b}(y,\;\Delta )=\delta y_{b}\left(1-\frac{z_{t2t1}}{2z_{a}}\Delta \right)e^{jk_{s}\left(z_{t2t1}\Delta +\frac{z_{at2}z_{t2}\Delta^{2}\tan^{2}\theta }{2z_{a}}\right)}f_{b}(y)\left[u_{in}\left(-\frac{z_{a}}{z_{b}}y\right)⊗E_{o}(y,\;\Delta )⊗h(y)\right],$$

(9) $$E_{o}(y,\;\Delta )=f_{a}(x_{0}+z_{t2}\Delta \tan\theta )E_{0}e^{jk_{s}\frac{x_{0}z_{at2}\Delta \tan\theta }{z_{a}}}D_{2I+1}\left(\frac{f}{\delta f_{FSR}}+\frac{z_{b}(x_{0}+z_{t2}\Delta \tan\theta )}{z_{a}\delta y_{b}}+\frac{y}{\delta y_{b}}\right),$$

(10) $$h(y)=\sum_{i=-I}^{I}A_{i}e^{j2\pi i\frac{y}{\delta y_{b}}},$$

where *D_N_*(*x*) represents the Dirichlet kernel [25], *f_b_*(*y*) is the image field of *u_b_*(*y’*) produced on the output coordinate *y* with the Fraunhofer diffraction, and *δy_b_* = *λz_b_*/(*n_s_d*). $g_{1}(y)⊗g_{2}(y)$ represents the convolution of the periodical functions *g*_1_(*y*) and *g*_2_(*y*) with a period of *δy_b_*.

The field at the output of the output slab illuminated from the single channel waveguide is broadened with the propagation in the output slab and expressed, using the Fourier transform of the mode field function of the single channel waveguide at the interface connected at position *y_s_* to the output slab, *u_s_*(*y’* − *y_s_*), as,

(11) $$E_{out}^{s}(y)=\sqrt{\frac{jk_{s}}{2\pi z_{b}}}E_{s}e^{-jk_{s}z_{b}}e^{j\frac{k_{s}y_{s}y}{z_{b}}}\int_{-\infty }^{\infty }u_{s}(y^{\prime })e^{j\frac{k_{s}yy^{\prime }}{z_{b}}}dy^{\prime },$$

where *E_s_* is the complex amplitude of the light at the end of the single channel waveguide. Consequently, the total field at the output of the output slab, *E_out_*(*y*, Δ), is given by:

(12) $$E_{out}(y,\;\Delta )=E_{out}^{b}(y,\;\Delta )+E_{out}^{s}(y).$$

### 3.4. Output Port

The complex amplitude of light coupled to, and output from, the *n*th output port, *t_n_*(Δ), is given by the overlap integral between *E_out_*(*y*, Δ) and the output mode field of the *n*th output waveguide *u_out_*(*y* − *y_n_*) as:

(13) $$t_{n}(\Delta )=E_{out}(y_{n},\;\Delta )*{\bar{u}}_{out}(-y_{n}).$$

where ${\bar{u}}_{out}$ represents the complex conjugate of *u_out_*. Using Equation (13), the output light from the AWG can be characterized.

### 3.5. Formation of Focusing Beams

For Methods (1) and (2), based on the quadrature detection of the phase and differential monitoring, four focusing beams are formed at the output edge of the output slab, using the waveguide array as a spatial filter as follows.

From Equation (11), the phase of the broad beam from the single channel waveguide at the edge of the output slab, *ϕ^s^*(*y*), is expressed as,

(14) $$\varphi^{s}(y)=\frac{\pi }{4}-k_{s}z_{b}+\frac{k_{s}y_{s}y}{z_{b}}+\varphi_{0}^{s},$$

where *ϕ^s^*_0_ is the phase shift by passing through the single channel waveguide. Allowing the phase and positon of the output port for monitoring the *m*th focusing beam at the edge of the output slab for a certain Δ to be *ϕ^b^_m_,* and *y^o^_m_*, respectively, the phase for the focusing beam relative to the broad beam monitored by the output port is given by *ϕ^b^_m_* − *ϕ^s^*(*y^o^_m_*). For example, when the phase for the second and fourth focusing beams is shifted from that of the first and third focusing beams by π/2 for the quadrature detection, *ϕ^b^_m_* must be set to satisfy the following relation:

(15) $$\varphi_{m}^{b}=\{\begin{array}{ll} \frac{k_{s}y_{s}y_{m}^{o}}{z_{b}}+\varphi_{0}\;\; & \left(m=1,\;3\right)\\ \frac{k_{s}y_{s}y_{m}^{o}}{z_{b}}+\varphi_{0}+\frac{\pi }{2} & \left(m=2,\;4\right) \end{array},$$

where *ϕ*_0_ is a constant.

The complex transmitting coefficient of the waveguide array, *A_i_*, is designed such that the four focusing beams, with the phases given by Equation (15), are formed from the waveguide array at the output edge of the output slab. Because the Fraunhofer diffraction image, proportional to the Fourier transform, of the output of the waveguide array at the output slab-array interface is produced at the output edge of the slab, the distribution of *A_i_* can be derived from the inverse Fourier transform of the field distribution of the desired focusing beams. If the amplitude field distributions of the four focusing beams have the same shape, *A_i_* is given by,

(16) $$A_{i}\propto \sum_{m=1}^{4}e^{j\left(\varphi_{m}^{b}-\frac{k_{s}idy_{m}^{f}}{z_{b}}\right)},$$

where *y^f^_m_* is the position of the *m*th focusing beam. In actuality, this phase and amplitude distribution can be obtained by adjusting the optical path length and insertion loss (e.g., the coupling loss between the slab-array interface) of each waveguide in the waveguide array.

## 4. Design Parameters

The performance of the proposed waveguide-based refractive index sensor was simulated using the theoretical model, described in Section 3. The design parameters are summarized in Table 1. We assumed the use of the silica-based PLC technology [15,16]. The relative index difference between the core and cladding was assumed to be 2.5% [26,27,28], which is a practical value using the silica-based PLC technology. The pitch *d* was set to 15 μm so that the coupling between the waveguides in the array could be negligible. The number of the waveguides in the waveguide array 2*I* + 1 was set so that sufficient power of the light from the input waveguide could be received at the array. Fundamental mode distributions, expressed as cos-exp functions, were used as the field distributions of the input/output waveguides at the interface to the input/output slabs because the cos-exp expression generally gives good approximation to the real field distributions in weakly-guided rectangular waveguides.

**Figure 3:**
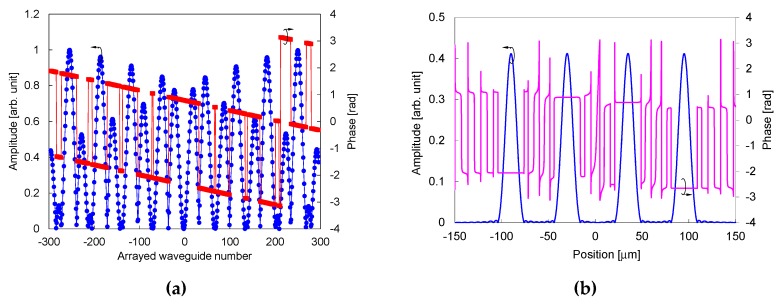
Design of waveguide array for spatial filtering. (**a**) Complex transmitting coefficient *A_i_*. (**b**) Resultant field distribution of focusing beams at edge of output slab from waveguide array.

The complex transmitting coefficient *A_i_,* calculated using Equation (16) and resultant field distribution of the focusing beams at the edge of the output slab, from the waveguide array, are plotted in Figure 3. Here, the refractive index of the material in the trench is assumed to be the same as the effective index of the slab. Four focusing beams are formed at positions −90, −30, 35, and 95 μm with the relative phases of zero (positions of −90 and 35 μm) and *π*/2 (positions of −30 and 95 μm) to the broad beam.

## 5. Simulation Results and Discussion

**Figure 4:**
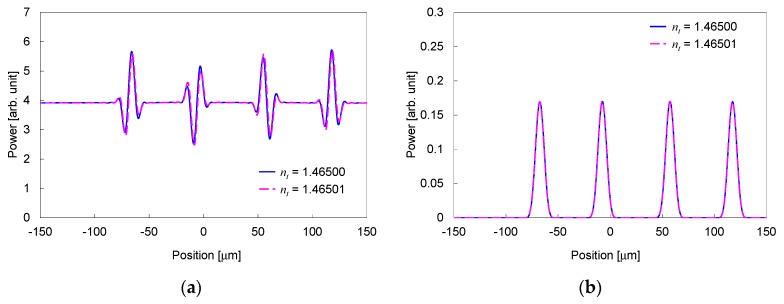
Power distribution at interface between output slab and output waveguides for two values of *n_t_* differed by 1 × 10^−5^. (**a**) Proposed structure. (**b**) Structure without broad beam.

Figure 4a shows the power distribution at the interface between the output slab and output waveguides for two refractive indices of the material under test *n_t_* differed by 1 × 10^−5^. For comparison, the optical power distribution without the broad beam, which corresponds to the power distribution for a conventional technique measuring the refraction angle, is indicated in Figure 4b. The focusing beams are located around the positions at −67, −7, 58, and 118 μm. In the proposed structure, shown in Figure 4a, power changes, due to the interference between the focusing beams from the waveguide array and broad beam, are observed around the positions of the focusing beams. A clear difference is observed among the power distributions for the proposed structure even when the difference of the refractive indices *n_t_* is as small as 1 × 10^−5^.

**Figure 5:**
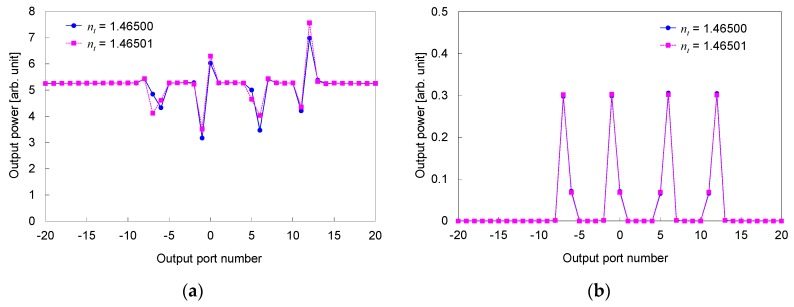
Output power from output ports for two values of *n_t_* differed by 1 × 10^−5^. (**a**) Proposed structure. (**b**) Structure without broad beam.

The output power from the output ports, corresponding to the measured power by the linear image sensor, is plotted in Figure 5a for two refractive indices *n_t_* differed by 1 × 10^−5^. The output power without the broad beam is shown in Figure 5b for comparison. The distributions of the output power clearly differ among the different indices for the proposed structure. Whereas, the distributions have no significant difference for the structure without the broad beam. This indicates that the proposed structure can detect a considerably smaller index difference, compared to the structure without the broad beam.

**Figure 6:**
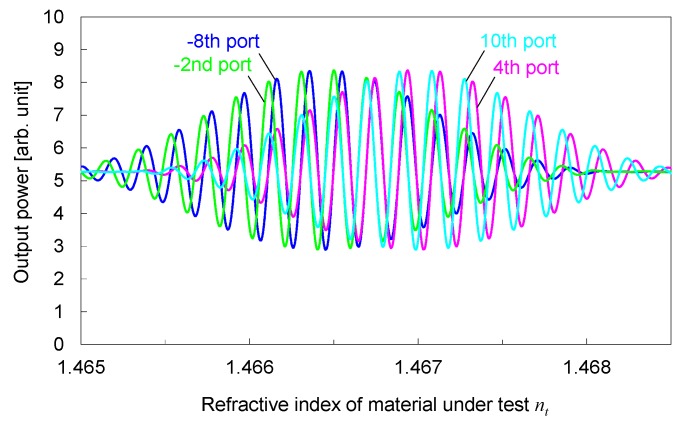
Output power from four output ports around focusing beams as a function of *n_t_*.

The output power from the four output ports around the focusing beams is plotted in Figure 6 as a function of the refractive index *n_t_*. The pairs of the –8th port/–2nd port and the 4th port/10th port have the relation of *π*/2 relative phase difference, described as Method (1) in Section 2. The pairs of the 4th port/–8th port and the 10th port/–2nd port have the in-phase relation described as Method (2). The output power from each port periodically changes as *n_t_* changes because the phase difference between the corresponding focusing beam and broad beam changes. The envelope of the periodical change is determined by the relative position of the focusing beam and output port. The closer the focusing beam and output port, the greater the magnitude of the envelope. For example, the magnitude of the envelope for the –8th port and –2nd port decreases whereas the magnitude of the envelope for the 4th port and 10th port increases as *n_t_* increases from about 1.4665 to about 1.4670. This indicates that each corresponding focusing beam is separating from the –8th port and –2nd port and, conversely, is approaching the 4th port and 10th port.

We observed that the output ports from which such index-dependent power changes are observed are shifted to the next ports when *n_t_* is shifted by approximately 1.1 × 10^−3^ RIU and the output ports are sequentially switched as *n_t_* changes per 1.1 × 10^−3^ RIU. This behavior can be understood because the Dirichlet kernel in Equation (9) is unchanged if *z_b_z_t_*_2_Δtan*θ/z_a_* + *y* is constant. Because the four focusing beams are ranged over 185 μm, which corresponds to the range of about 19 channels of the output ports, when *N* output ports are arranged, the refractive index range of roughly (*N* − 19) × 1.1 × 10^−3^ RIU can be measured.

**Figure 7:**
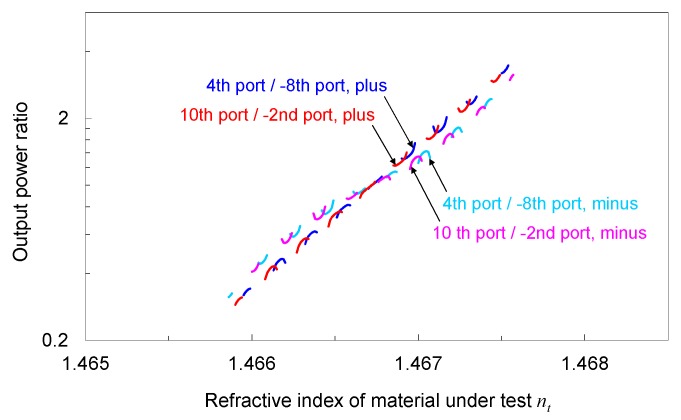
Output power ratio for 4th port/–8th port and 10th port/–2nd port as a function of *n_t_*. The power ratio is calculated using the relative output power, i.e., the difference between the output power and power if only the broad beam exists.

The magnitude of the envelope of the output power depends on the refractive index *n_t_,* such that *n_t_* can be roughly estimated from the magnitude of each output port. However, the magnitude of the envelope cannot be determined from the output power for only one output port because the output power depends, not only on the magnitude, but also on the phase difference between the focusing beam and broad beam. The ratio of the magnitude for the two output ports can be determined by the ratio of the output power from the output ports if these ports have an in-phase relation. As per Method (2), the focusing positions, depending on *n_t_*, can be roughly estimated by comparing the output power from the output ports that have an in-phase relation. Hence, in the proposed method, *n_t_* is roughly estimated by calculating the ratio of the output power from two output ports that have an in-phase relation and using the relation between the ratio and *n_t_*. Figure 7 plots the output power ratio for the 4th port/–8th port and 10th port/–2nd port. Here, the power ratio is calculated using the relative output power, i.e., the difference between the output power, and power if only the broad beam exists. In Figure 7, the difference in the sign of the relative output power is represented by different lines. The data for the relative output power close to zero are neglected from the plots. The power ratio tends to increase as *n_t_* increases. Each group of continuous lines is well separated from the others in terms of the power ratio over the range greater than 1.1 × 10^−3^ RIU (*n_t_* of approximately 1.4660–1.4675). This indicates that one can determine what group of continuous lines to which the calculated power ratio belongs. Because each group of continuous lines has a different range of *n_t_*, *n_t_* can be roughly estimated from the power ratio.

**Figure 8:**
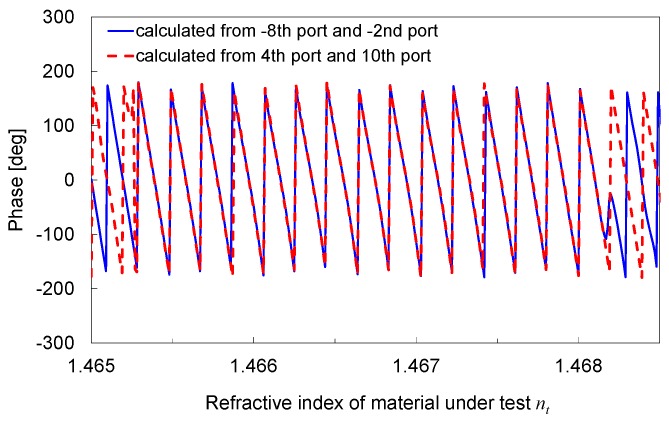
Phase calculated from –8th port/–2nd port and 4th port/10th port as a function of *n_t_*.

The phase calculated from the –8th port/–2nd port, and that calculated from the 4th port/10th port, are shown in Figure 8. The phase monotonically increases and periodically changes as *n_t_* increases over the refractive index range greater than 1.1 × 10^−3^ RIU. Thus, *n_t_* can be precisely estimated by two steps, i.e., *n_t_* is first roughly determined as the range of *n_t_* where the power ratio belongs to the corresponding group of the continuous lines, as shown in Figure 7, and then precisely determined from the phase within this *n_t_* range.

**Figure 9:**
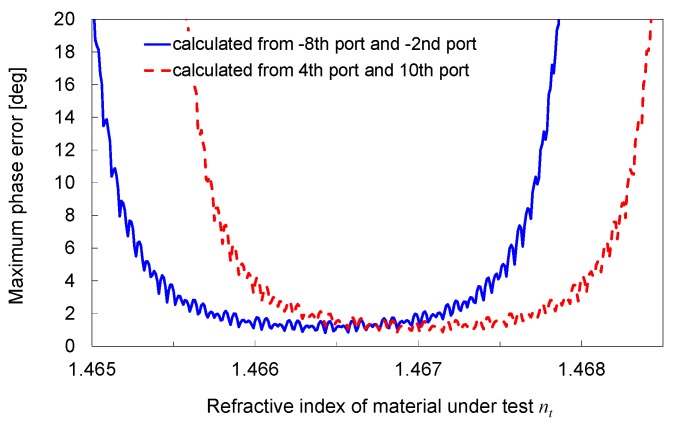
Deviation of phase calculated from –8th port/–2nd port and 4th port/10th port under condition that monitored output power is deviated by ±0.05 dB.

To estimate the error in *n_t_* due to the error in the monitored output power in measurement, we calculated the deviation in the phase assuming that the monitored output power is deviated by ±0.05 dB (corresponding to approximately a 1% error). Figure 9 plots the phase deviation calculated for the same pairs of the output ports, as indicated in Figure 8. The phase deviation tends to increase as the absolute value of the relative output power decreases. Because the two values of the phase are calculated from the two pairs of the output ports, the phase value with the smaller error can be determined by selecting the pair with the grater absolute value of the relative output power. The maximum phase error within the *n_t_* range of 1.1 × 10^−3^ RIU (*n_t_* = 1.4662–1.4673) is 1.59°. Because the phase sensitivity is 1.57–2.27 × 10^6^/RIU within this *n_t_* range, this phase error corresponds to an error in *n_t_* of 0.70–1.01 × 10^−6^ RIU. This result indicates that the error in *n_t_* can be suppressed to approximately 1 × 10^−6^ RIU or less, even if the monitored power deviation of ±0.05 dB is accepted. Related to this result, it is preferable to use a linear image sensor, with good repeatability, in a real device because the deviation in the monitored power by the linear image sensor directly affects the error in the estimated refractive index *n_t_*.

In this paper, a simulation was performed using the design parameters, shown in Table 1. Better performance could be expected by optimizing the design parameters. For example, the sensitivity of the refractive index could be improved by optimizing the relations between the opening angle of the trench *θ* and pitch of the output waveguides at the interface to the output slab, because the amount of the shift of the focusing beams, according to the change in *n_t_* depends on *θ*.

## 6. Conclusions

The proposed transmission-type refractive index sensor, based on PLC technology, can potentially provide high precision refractive index sensing using a compact integrated device. A combination of coarse measurements based on the dependence of the refraction angle, and fine measurement based on the dependence of the phase on the refractive index of the material under testing, was introduced to measure the absolute refractive index precisely, without expensive optical measurement equipment, such as an OSA. The simulation results suggest that a refractive index error of approximately 1 × 10^−6^ RIU or less can be theoretically expected using the proposed method. In this paper, we simulated the performance using a circuit modeling based on Fourier optics and transfer function. As a future work, it would be desirable to verify the results through other simulation methods, such as the beam propagation method (BPM) and evaluate the performance, including real conditions such as the tolerance to fabrication error. Moreover, the fabrication of waveguide devices and an experiment would be needed to evaluate the actual performance. The proposed method could be attractive for the precise refractive index measurements of liquids in the biochemical, environmental, or industrial fields.

## Figures and Tables

**Table 1:** Design parameters.

Description	Parameter
Wavelength *λ*	1.55 μm
Relative index difference between core and cladding	2.5%
Core width of input waveguide at the interface to input slab	9 μm
Core width of output waveguides at the interface to output slab	2 μm
Pitch of output waveguides at interface to output slab	10 μm
Spacing of focusing beam pair for phase estimation	60 μm
Spacing of focusing beam pair for estimation of beam position	125 μm
Pitch of waveguide array at interface to input/output slabs *d*	15 μm
Number of waveguides in waveguide array 2*I* + 1	593
Focal length of input slab *z_a_*	30 mm
Focal length of output slab *z_b_*	60 mm
Distance between input waveguide and trench *z_t_*_1_	16 mm
Propagation length of trench *z_t_*_2*t*1_	8 mm
Wedge opening angle of trench *θ*	15°
